# WIN-MTB-2024002 – WIN International Molecular Tumor Board: A 52-year-old female with unmeasurable Stage IIIc colon cancer

**DOI:** 10.18632/oncotarget.28921

**Published:** 2026-10-16

**Authors:** Wafik S. El-Deiry, Alberto Hernando-Calvo, Shai Magidi, Catherine Bresson, Sadakatsu Ikeda, Hikmat Abdel-Razeq, Arjun Oberoi, Susana Aguilar Izquierdo, Zhihuang Hu, Tobias Meissner, Rachel Elsey, Amal Al-Omari, Fanny Wunder, Razelle Kurzrock

**Affiliations:** ^1^Worldwide Innovative Network (WIN) Association - WIN Consortium, Chevilly-Larue 94550, France; ^2^Legorreta Cancer Center at Brown University, Providence, Rhode Island 02912, USA; ^3^Vall d'Hebron Hospital Campus and Institute of Oncology (VHIO), Barcelona 08035, Spain; ^4^Fudan University Shanghai Cancer Center, Shanghai 200032, China; ^5^Institute of Science Tokyo Hospital Tokyo 113-8510, Japan; ^6^King Hussein Cancer Center, Amman 11941, Jordan; ^7^Avera Cancer Institute, Sioux Falls, South Dakota 57105, USA; ^8^Medical College of Wisconsin, Milwaukee, Wisconsin 53226, USA; ^*^These authors contributed equally to this work

**Keywords:** cancer, precision oncology, molecular tumor board, colon cancer

## Abstract

Colorectal cancer (CRC) is a leading cause of cancer-related mortality, with a complex molecular profile that impacts tumor behavior and treatment response. This case report discusses a 52-year-old female diagnosed with Stage IIIc colon cancer, exhibiting *KRAS*, *TP53*, *IDH1*, and *APC* mutations, as well as a tumor microenvironment with high PD-L1 expression and elevated VEGFR1 and VEGFR3. Despite surgical resection and adjuvant treatment which included FOLFOX, FOLFIRI with bevacizumab and local radiation, the patient’s disease was difficult to track due to small, non-FDG-avid lymph nodes in the aortocaval region and persistent circulating tumor DNA (ctDNA). Following a review by an international Molecular Tumor Board (MTB), maintenance therapy options were discussed, including the personalized combinations of regorafenib, sulindac, and metformin, or fruquintinib, sulindac, and metformin, based on the patient’s molecular alterations. Alternatively, 5-FU with or without bevacizumab was suggested. In the event of relapse, the MTB recommended the combination of regorafenib, sulindac, and metformin, with the addition of trametinib and ivosidenib. The combination of regorafenib, ipilimumab, and nivolumab (RIN) was also considered due to the patient’s high PD-L1 expression. Further clinical trial options, such as pan-RAF inhibitors, NK cell agonists, and TAS-102 with bevacizumab, were also discussed. Additionally, the potential combination of PD-1 and TGFβ inhibitors was explored. This case underscores the critical role of molecular profiling in tailoring personalized treatment strategies for advanced CRC, providing novel avenues for managing recurrent disease, even in the absence of measurable disease.

## INTRODUCTION

Colorectal cancer (CRC) is one of the most prevalent cancers worldwide, with over 2.06 million new cases diagnosed annually, making it a leading cause of cancer-related mortality globally [1]. CRC is a highly heterogeneous disease, characterized by a complex molecular landscape that includes a diverse range of genetic mutations, epigenetic alterations, and dysregulated signaling pathways [2–5]. These molecular alterations significantly influence tumor behavior, prognosis, and responses to therapeutic interventions [3, 6–8]. Molecular profiling has emerged as an essential tool in understanding CRC pathogenesis, revealing critical pathways that shape tumor behavior and providing insights into personalized treatment strategies [5, 9–11].

*KRAS* mutations are commonly implicated in CRC and constitute activation of the MAPK/ERK and PI3K/AKT pathways, promoting cell proliferation and resistance to targeted therapies [12, 13]. Mutations in *TP53*, also highly frequent in CRC, lead to the loss of its tumor-suppressive functions, disrupting apoptosis and cell cycle regulation [14–16]. Mutations in *APC* disrupt its role as a negative regulator of Wnt signaling, leading to constitutive activation of the pathway, which is a hallmark of CRC pathogenesis [17]. Although rare in CRC, *IDH1* mutations are recognized as drivers in the early stages of tumorigenesis [18]. The immune microenvironment also plays a pivotal role in CRC progression [19]. High PD-L1 expression is associated with immune evasion, while elevated vascular endothelial growth factor receptors (VEGFR) promote angiogenesis and tumor progression [20, 21].

Stage IIIc colon cancer, a subtype of CRC, is characterized by the spread of cancer to regional lymph nodes, and requires a multidisciplinary treatment approach, including surgical resection of the primary tumor followed by adjuvant chemotherapy to reduce the risk of recurrence. Despite surgical intervention and chemotherapy, the risk of recurrence remains significant, particularly when lymph node involvement is extensive [22, 23]. Monitoring for recurrence in these patients can be particularly challenging, especially when lymph node metastases are not easily detectable or measurable using conventional imaging techniques [24]. Lymph node metastasis is a critical prognostic factor in colon cancer, with increased lymph node involvement correlating with a higher risk of disease recurrence and poor survival outcomes [25, 26]. In many cases, even after resection of the primary tumor, small or indeterminate lymph node metastases may persist, remaining difficult to detect or accurately measure using imaging modalities, such as computerized tomography (CT) or positron emission tomography (PET) scans. This creates a dilemma in patient management, as traditional imaging may fail to identify the recurrence of disease, making it difficult to determine the most appropriate course of action for treatment.

This report describes the clinical course and international Molecular Tumor Board (MTB) discussion of a 52-year-old female patient diagnosed with stage III colon cancer who presented with a unique molecular profile of *KRAS*, *TP53*, *IDH1*, and *APC* mutations alongside a tumor microenvironment with high PD-L1 expression, elevated levels of VEGFR1 and VEGFR3, and active immune natural killer (NK) cell infiltration. The patient’s disease was difficult to track due to low fluorodeoxyglucose (FDG) uptake in unresectable lymph nodes and their small size on imaging. An international MTB, comprising expert physicians and scientists specializing in personalized cancer medicine from 18 centers across 13 countries, as shown in Figure 1, convened by the Worldwide Innovative Network (WIN) Association - WIN Consortium, reviewed and discussed the case. Although the patient had no measurable disease as assessed by imaging at the time of reviewing the case, the board discussed potential therapeutic options for maintenance and in the event of disease relapse. This report extends prior published case-based discussions from the WIN Consortium’s International MTB [27].

**Figure 1:**
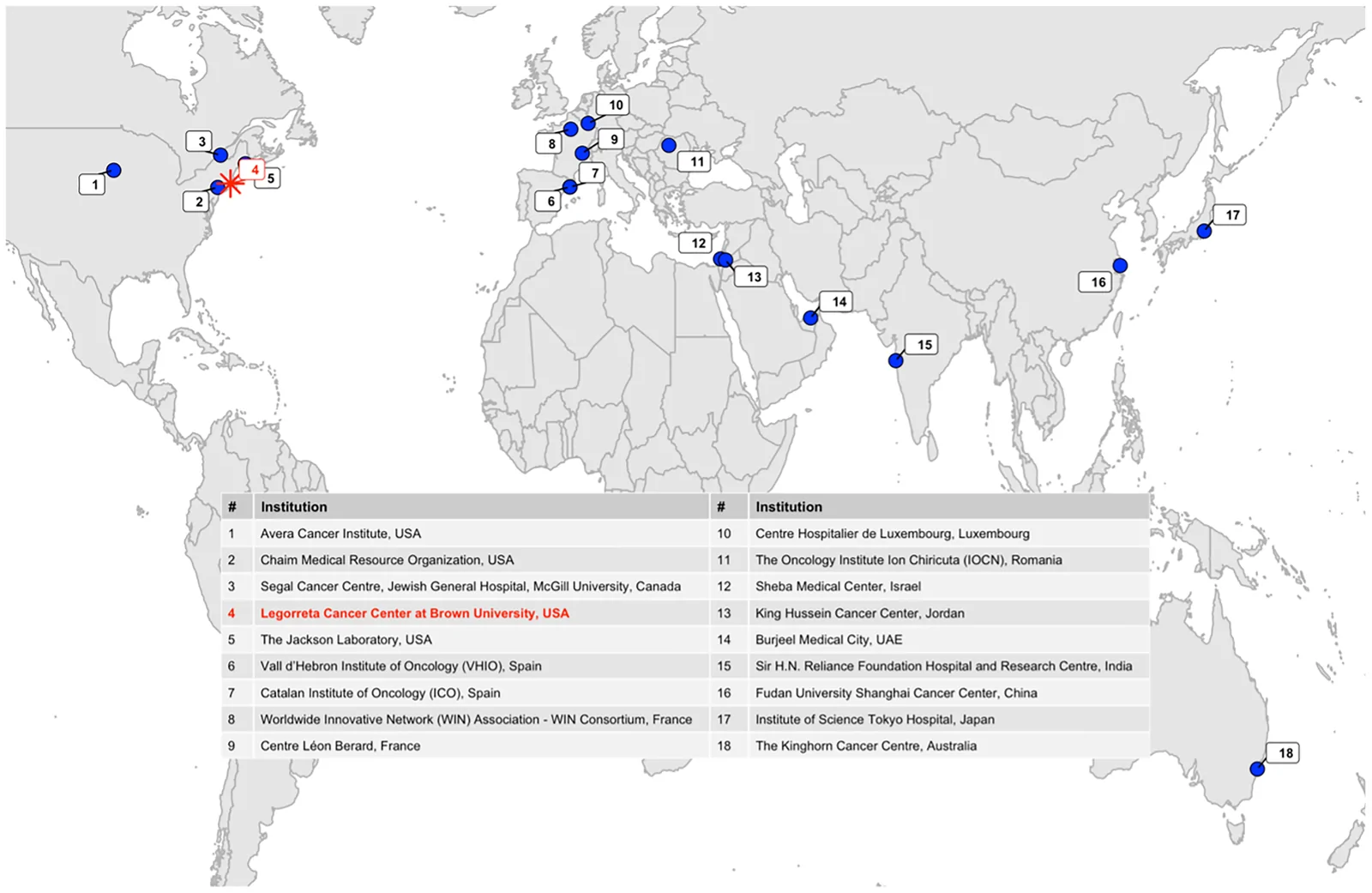
WIN Consortium International Molecular Tumor Board. The figure highlights 18 institutions participating from countries worldwide (blue dots), with the patient discussed in the MTB originating from USA (red asterisk).

## CASE REPORT DESCRIPTION

The patient is a 52-year-old woman who was diagnosed with colon cancer. She has a family history of cancers: her father had melanoma, three uncles died from cancer of unknown origin on her father’s side, her sister passed away from breast cancer at the age of 37, and multiple relatives on her mother’s side have had colon cancer. Figure 2 outlines the patient’s treatment course and molecular testing timeline.

**Figure 2:**
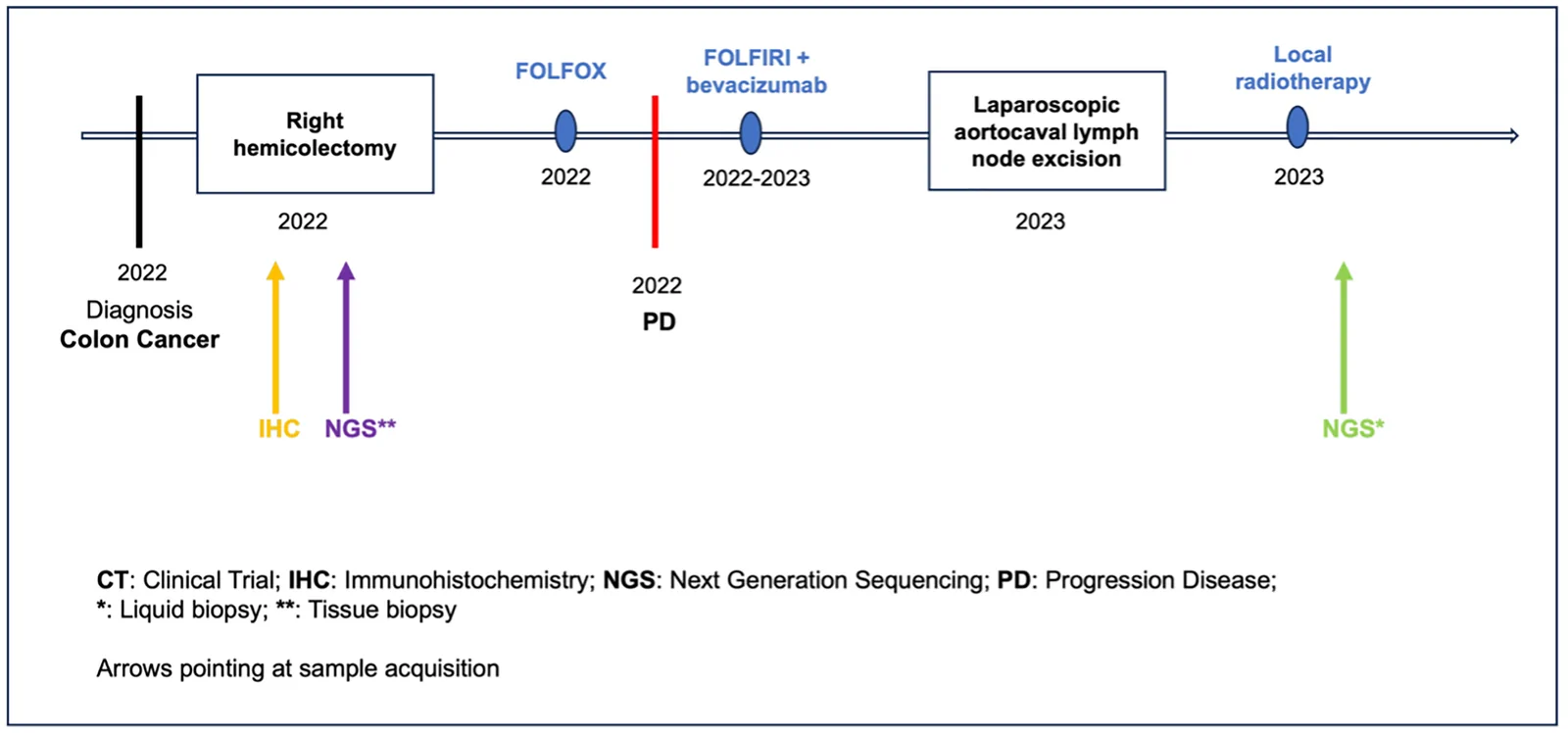
Treatments and molecular tests timeline. The figure outlines the treatments and molecular tests the patient has undergone throughout her medical history.

In June 2021 she began experiencing symptoms, including unexplained weight loss, paleness, and loss of appetite. In February 2022, her hemoglobin test, done by her primary care physician, revealed a critically low level of 5.9 g/dL, prompting hospitalization where she received five units of packed red blood cells (PRBCs) transfusions and iron supplementation. Later that month, she underwent a colonoscopy, which revealed a completely obstructing, friable, ulcerated mass in the right lower quadrant near the groin region, along with an 8 mm polyp in the proximal transverse colon.

Shortly after the procedure, she underwent a right hemicolectomy. Her tumor was classified as pT4bN2bM0 stage IIIc. The pathology report described the tumor as a moderately differentiated adenocarcinoma with mucinous features, in the size of 6 × 6 × 3.5 cm, invading the muscularis propria into the pericolonic tissue and focally into the omentum, which made the classification of pT4b. Additionally, the tumor had extended into the terminal ileum wall with evidence of lymphovascular invasion. Ten out of 25 lymph nodes were positive for metastatic adenocarcinoma, marking the tumor as pN2b. Tumor deposits were present, but the margins of resection were negative for tumor. Tumor histology is shown in Figure 3. Immunostaining showed that the cells were positive for CDX2 and SATB2, consistent with colorectal origin. Immunostaining for NTRK was negative, as well as for HER2 which was negative with a score of 0.

**Figure 3:**
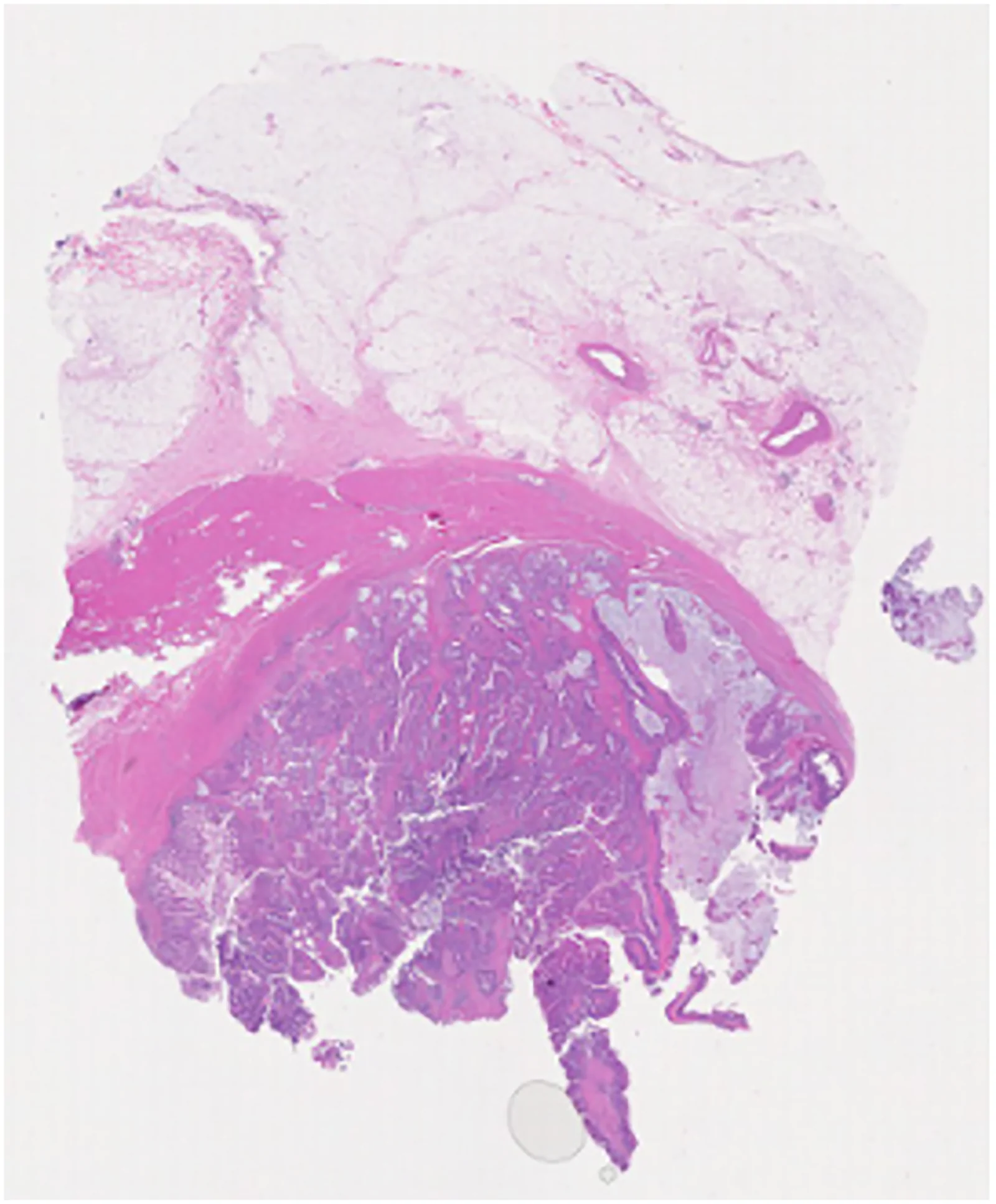
Histopathological features of the resected colon tumor. Hematoxylin and eosin staining demonstrates moderately differentiated adenocarcinoma with mucinous features. Annotations indicate representative tumor areas and mucinous components. Immunohistochemistry performed by the treating institution showed tumor cells positive for CDX2 and SATB2, consistent with colorectal origin, and negative NTRK and HER2.

In March 2022, following surgery, the patient started adjuvant chemotherapy with FOLFOX, completing six months of treatment. A CT scan in September 2022 showed no evidence of recurrence, although a 6 mm left-para-aortic lymph node and an 8 mm left lobe of liver hypodensity were noted. A follow-up CT scan in October 2022 showed no changes in the size of the 6 mm left-para-aortic lymph node or the 8 mm left lobe of liver hypodensity, while it was considered possibly a small liver cyst. However, new findings from the scan identified three new abnormal-appearing retroperitoneal and mesenteric root lymph nodes – a 1.6 × 1.7 cm aortocaval lymph node, a 1.2 × 1.2 cm right mesenteric root lymph node, and an adjacent 11 × 9 mm right pericaval lymph node. These findings raised suspicion for tumor recurrence.

The patient underwent multiple molecular profiling tests using both liquid and tissue biopsies, as outlined in Table 1 and Figure 4. The FoundationOne tissue analysis available in November 2022 showed that the tumor was microsatellite stable (MSS), with a tumor mutational burden (TMB) of 5 mutations per megabase. Genomics findings included *KRAS*, *IDH1* and two *APC* mutations. In addition, an in-frame deletion in *PIK3CA*, an insertion in *PIK3R1*, *TP53* G245S and two *SOX9* mutations were identified. In response to these new findings, the patient began combination treatment of FOLFIRI and bevacizumab, completing 11 cycles of this regimen, which continued until March 2023. CT scan of the chest, abdomen, and pelvis (CT-CAP) done after completing the treatment showed minimal changes in the small retroperitoneal and mesenteric lymph nodes.

**Table 1:** Molecular alterations

Date of testing		November/2022	September/2023	December/2023	December/2023
Date of sample acquisition		February/2022	February/2022	December/2023	December/2023
**Sample**		Tissue (right hemicolectomy)	Tissue (right hemicolectomy)	Blood	Blood
**Laboratory**		FoundationOne	BostonGene (Comprehensive Genomic Profiling)	Guardant (Gaurdant360)	GRAIL (Galleri)
**TMB (mutations/mb)**		5	3.09	6.33	
**Microsatellite Status**		Stable	Stable	Stable	
**HRD (positive with value; equivocal with value; negative with value/not done (ND))**					
**LOH (positive with value; equivocal with value; negative with value/not done (ND))**					
**Mutations (MAF)**	TP53 Splice Site SNV			+ VAF = 0.4%	
	NRAS	WT			
	KRAS G12S	+	+ GOF		
	IDH1 R132C	+	+ GOF, VAF = 15.4%		
	TP53 G245S	+	+ LOF, VAF = 8.7%		
	APC R805*	+	+ LOF, VAF = 18.2%		
	APC L1488Yfs*19, R805*	+	+ LOF, VAF = 14.9%		
	PIK3CA C420-P421del	+			
	PIK3R1 R461_E462insVR	+			
	SOX9 E226fs*26	+			
	SOX9 H380fs*4	+			
**Amplifications**					
**IHC tests**	PD-L1		High		
**Other findings**	Tumor Microenvironment		· Fibrotic microenvironment · High PDL1 expression · Immune active infiltrate with high number of NK cells · Medium level of immune suppressive infiltrate · High expression of VEGFR1 and VEGFR3		
	RNA-Seq		Low HER2 expression, CMS4		
	DNA methylation patterns				DNA methylation patterns that are often associated with cancer
	Germline alterations		· No HRR gene · No DPYD germline alterations · No germline alterations associated with risk of colorectal cancer		

Data was provided where available. Only pathogenic alterations are included, and variants of unknown significance (VUS) are not included. Abbreviations: CMS: consensus molecular subtype; GOF: gain of function; HRD: homologous repair deficiency; HRR: homologous recombination repair; IHC: immunohistochemistry; LOF: loss of function; LOH: loss of heterozygosity; NK: naturel killer; TMB: tumor mutational burden; VAF: variant allele frequency; WT: wild type.

**Figure 4:**
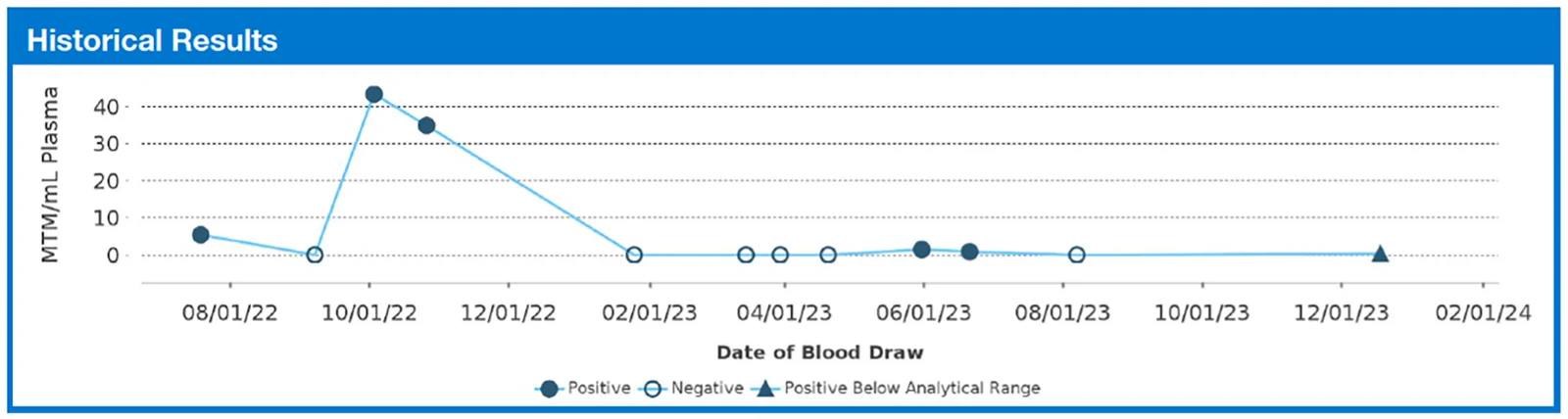
Signatera results from 2022 and 2023. The figure outlines the mean tumor molecules per milliliter (MTM/mL) in the blood showing measurable ctDNA and suggesting presence of residual disease.

In May 2023, the patient underwent a diagnostic laparoscopy with biopsy, which involved laparoscopic mobilization of the duodenum, ureterolysis, and excision of aortocaval lymph nodes. Biopsies from the pelvic sidewall and three aortocaval lymph nodes were all negative for cancer.

By June 2023, a PET-CT scan was performed. Comparison to a prior scan from November 2022, done following the initial surgery, showed decrease in size but increase in FDG uptake in the lymph nodes in the aortocaval area, suspicious for nodal metastasis. The mesenteric nodes were not FDG-avid and appeared stable.

In August 2023, the patient underwent five treatments of local radiotherapy to the suspicious aortocaval lymph nodes. Detailed information regarding the radiotherapy technique and dose/fractionation was not available. Subsequent PET-CT scan done in September 2023 showed a decrease in both size and FDG uptake of the previously suspicious aortocaval lymph nodes. The mesenteric nodes, which were not FDG-avid, appeared stable as shown also in the previous scan. The small left liver lobe cyst was stable as well.

Molecular analysis by BostonGene’s Comprehensive Genomic Profiling on tissue from the hemicolectomy, which was available in September 2023, classified the tumor as Consensus Molecular Subtype 4 (CMS4). The analysis confirmed previous findings, including a gain-of-function *KRAS* (G12S) mutation, loss-of-function of *TP53* mutation (G245S), a gain-of-function *IDH1* mutation (R132C), and two loss of-function mutations in *APC* (R805* and L1488Yfs*19). The tumor microenvironment was characterized by fibrosis, high PD-L1 expression, active immune infiltration by natural killer (NK) cells, and elevated expression of *VEGFR1* and *VEGFR3*. No mutations were found in *BRAF*, *DPYD*, *EGFR*, *HER2*, *MET*, *NRAS*, *NTRK1/2/3*, *PIK3CA*, *POLE* or *PTEN*. Additionally, no germline alterations related to homologous recombination or those associated with an increased risk of colorectal cancer were detected.

Another molecular profiling test was performed on the blood using the Guardant360 test in December 2023, which revealed persistent circulating tumor DNA (ctDNA). The Guardant report identified several findings, including a *TP53* splice variant and other variants of unknown significance (VUS) in several genes. Consistent with previous tests, the patient’s tumor was MSS, with a low TMB of 6.3 mutations per megabase.

Carcinoembryonic antigen (CEA) levels in the blood have been monitored regularly from March 2022 to December 2023 to assess treatment response and detect recurrence. Overall, the CEA levels have generally remained low, with the highest recorded value being 11.97 ng/mL in October 2022. This increase coincided with the patient’s diagnosis of aortocaval nodal involvement and the initiation of the FOLFIRI plus bevacizumab treatment. By August 2023, following the local radiation therapy, CEA levels returned to normal. Although the CEA levels were elevated on a few occasions, with 6 ng/mL in August 2022 and 11.97 ng/mL in October 2022, most measurements were 5 ng/mL or lower, with the most recent reading in December 2023 being 3.1 ng/mL. This pattern suggests that the local radiation therapy has been effective for the patient.

The patient also underwent multiple Signatera tests in 2022 and 2023, as shown in Figure 4, to detect mutated tumor DNA in the blood. Although the levels of ctDNA were low, they were still measurable, suggesting the presence of residual disease. These findings align with the results from the Guardant360 test, which also continues to show measurable ctDNA.

Moreover, the patient had subsequent GRAIL Galleri tests between September and December of 2023 to detect cell-free DNA (cfDNA) released by cancer cells into the bloodstream. The last two test results detected DNA methylation patterns that are often associated with cancer. These positive results indicating the presence of epigenetic changes consistent with cancer, although no specific mutations were identified.

Another PET scan performed in January 2024 showed further resolution of the lymph nodes that had been previously highlighted. While in the June 2023 scan, there was some FDG uptake in the aortocaval nodes, it was no longer visible in January 2024, as shown in Figure 5. The comparison of the two PET-CT scans indicated that the previously suspicious areas no longer exhibited FDG activity, suggesting the absence of active disease. There were no signs of FDG avidity in the chest, liver, or abdomen in either scan.

**Figure 5:**
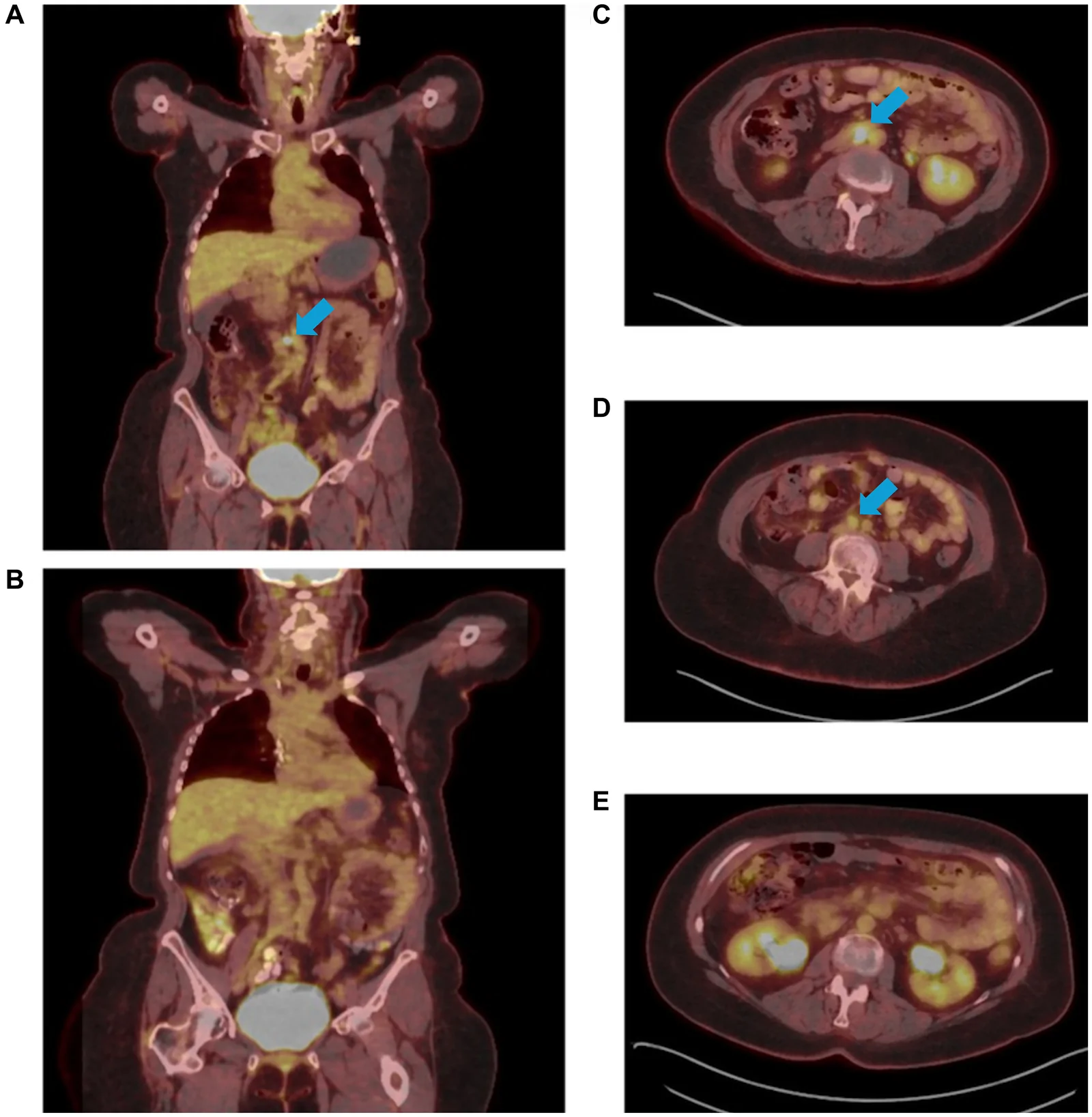
PET-CT scans showing changes in the aortocaval lymph nodes between from 2023 and 2024. Arrows indicate the area of suspicious FDG uptake in the aortocaval nodes on the June 2023 PET-CT (**A**, **C**, **D**). The corresponding regions on the January 2024 PET-CT images show subsequent resolution, with no visible FDG uptake (**B**, **E**).

While the patient’s most recent treatment was local radiotherapy in August 2023, the disease status remained clinically challenging to define. The January 2024 PET-CT showed no FDG-avid disease, and prior biopsies of the pelvic sidewall and aortocaval lymph nodes were negative for cancer. However, consecutive positive ctDNA results by Signatera and Guardant360 raised concern for molecular residual disease despite the absence of measurable disease on conventional imaging. Therefore, at the time of MTB discussion, the patient was best characterized as having no radiographically measurable disease, but suspected molecular residual disease based on persistent ctDNA positivity. The patient was not receiving active systemic therapy, and discussions were ongoing regarding potential maintenance strategies and management options if radiographic or clinical progression occurred.

## DISCUSSION

The WIN International Consortium MTB convened to discuss potential next steps in the setting of no radiographically measurable disease, but suspected molecular residual disease based on persistent ctDNA positivity. One key consideration was whether the patient should undergo further chemotherapy, taking into account the presence of ctDNA and the time lapse since the last therapy, with surgery being considered if the lymph nodes become resectable. In such a case, it was highlighted that surgery would be followed by additional adjuvant therapy, depending on the tumor’s characteristics and available data. Another important point for the MTB was determining the approach in the event of progression. The MTB discussed potential therapies based on the patient’s molecular profile. However, treatment decisions are typically made when there is a measurable disease to monitor. It was noted that the original tumor was pT4bN2b with numerous positive lymph nodes, indicating a high risk of relapse. Since the patient does not have easily trackable disease, the focus of the discussion was on adjuvant therapy, both as maintenance and in the event of progression. The therapeutic approaches discussed below should therefore be interpreted as MTB-generated, molecularly informed considerations. Some may be clinically accessible in selected settings, including through approved indications, off-label use, or clinical trials. However, because post-MTB treatment and follow-up are not documented in this report, the discussion is intended to inform therapeutic reasoning rather than to provide evidence of patient-level outcome from any specific approach. For maintenance therapy, considering the patient’s current situation where disease is not easily followed, a combination of regorafenib, sulindac and metformin could be considered. Regorafenib is a VEGFR inhibitor typically used in third-line therapy. Some data suggest that regorafenib may also inhibit the Wnt pathway [28], which is particularly relevant given the patient *APC* alterations. As for the *KRAS* mutation, while regorafenib inhibits RAF, it is not expected that it will directly affect the RAS pathway in a way that would either benefit or harm the patient. For these reasons, regorafenib would be a viable option. While regorafenib is typically not used in the adjuvant setting due to its toxicity, it was noted that physicians may be reluctant to follow this approach. However, clinical experience suggests that by substantially reducing the dose, regorafenib’s tolerability would likely improve. It was recommended that when using regorafenib in combination with other therapies, its dose should be reduced to half – for example, 80 mg with 3 weeks on and 1 week off. Sulindac, a Wnt pathway inhibitor, could complement regorafenib’s effect given that *APC* activation is part of the Wnt pathway. Metformin, an mTOR pathway inhibitor, could be considered given the patient’s *PIK3R1* alteration. Both sulindac and metformin have been shown to be well-tolerated in patients. Maximizing biomarker-matching to molecular alterations correlates with improved outcomes [29–32].

Fruquintinib, a VEGFR1 and VEGFR3 inhibitor, was also mentioned as a possible option [33–35]. If considered, pairing it with sulindac and metformin would be favored. However, regorafenib was discussed as potentially being more advantageous based on the patient’s molecular profile. Interestingly, FRESCO-2 [33], the fruquintinib trial, included patients who had previously failed regorafenib therapy, which could provide further insight into its potential efficacy.

Another option that was discussed as a maintenance therapy included 5-FU alone or in combination with bevacizumab, an anti-VEGF. This option could also be considered in the neoadjuvant setting if the lymph nodes become resectable.

In the event of relapse, it was discussed that in addition to performing another biopsy, the combination of regorafenib, sulindac and metformin would still be suggested, with the addition of trametinib to target the *KRAS* alterations and ivosidenib to target *IDH* mutations. Individual components or selected combinations discussed by the MTB have been investigated clinically. However, the safety and efficacy of the proposed multi-agent regimen have not been established.

Therefore, the discussion included starting with lower doses (half doses) and closely monitoring the patient on a weekly basis through clinic visits and laboratory tests, including complete blood count with differential, platelet count and metabolic profile, to assess toxicity and determine whether dose adjustments are necessary. It was mentioned that the combination of a MEK inhibitor, such as trametinib, with PIK3CA pathway inhibitor that is an anticancer drug is expected to be toxic. However, metformin can be combined safely with MEK inhibitors.

Clinical trials could also offer alternative treatment options if relapse occurs, particularly those investigating pan-RAF inhibitors. Tovorafenib, among other agents, was mentioned as having been investigated in clinical trials and as a potential avenue to explore [36].

Another regimen discussed was regorafenib, ipilimumab, and nivolumab (RIN). This approach could be considered if the patient progresses, especially given the patient’s high PD-L1 expression levels.

It was also discussed that whilst CMS4 is not a definitive biomarker for treatment decisions, it is associated with increased TGFβ and potentially Wnt/β-catenin activation [37], which aligns with the previous discussion on treatment options. If the patient progresses, combining a PD-1 inhibitor with a TGFβ inhibitor could be considered, though are not commonly used in clinical trials and not yet approved. Nonetheless, this remains a potential consideration.

From an immunotherapy perspective, the patient’s tumor demonstrated high infiltration of NK cells, which opens the possibility of exploring clinical trials, despite the patient being MSS. While the phase I clinical trial (NCT05277051) investigating GSK4381562-based immunotherapy combinations in selected advanced solid tumors, including colorectal cancer, in the USA, Europe, Japan, Australia, China and Canada is no longer enrolling new patients, exploring other active studies targeting NK cell-directed immunotherapy pathways could be a valuable consideration for the patient.

In addition, data from the SUNLIGHT study regarding the combination of TAS-102 and bevacizumab was mentioned. The study results showed that when combined with bevacizumab, TAS-102 extended overall survival [38]. This makes the combination of TAS-102 with bevacizumab a reasonable treatment option to consider.

Antibody-Drug Conjugates (ADCs) were also discussed, but it was determined that they were not an appropriate therapy for this patient at this time. ADCs are directed against specific surface antigens (e.g., ER, AR, PD-1, PDL-1, HER2, Claudin 18.2, FOLR1, NECTIN4, TROP2, LAG3 as detected by immunohistochemistry (IHC)), but the relationship between antigen expression level and clinical activity can be variable [39]. In this case, the patient was negative to HER2 by IHC, making a HER2-directed ADC strategy less compelling. ADCs are effective due to their payloads, and while it may not be relevant at this moment, they could become an option if the patient relapses. However, it would be worthwhile to perform an IHC panel to further assess the patient’s potential eligibility for ADCs in the future.

It was also noted that for patients with normal or low CEA levels, as in this case, aggressive approaches may not be necessary. Instead, a single-agent maintenance therapy, such as capecitabine, could be an appropriate option for this patient or 5-FU for a period of time, followed by surveillance.

It was also mentioned that regular use of aspirin after diagnosis was associated with longer survival among patients with mutated-PIK3CA colorectal cancer, but not among patients with wild-type PIK3CA cancer [40, 41].

Consistent with the educational scope of this MTB series, this report describes the molecular interpretation and therapeutic considerations generated by the international MTB, rather than the clinical outcome of an implemented MTB-guided intervention. At the time of MTB review, the patient had no measurable disease on conventional imaging and was not receiving active systemic therapy, while management was complicated by concern for residual disease based on persistent ctDNA positivity. The report does not include implementation of a specific MTB-recommended strategy or subsequent post-MTB outcome data. Therefore, the MTB discussion was intended to illustrate, for educational purposes, precision oncology decision-making regarding possible maintenance strategies and therapeutic options in the event of disease relapse, rather than to document the outcome of a specific treatment intervention. Further clinical follow-up will be required to determine whether an MTB-informed strategy is implemented and whether it affects patient outcome.

This case highlights the role of international MTBs in supporting complex therapeutic decision-making when conventional imaging, tissue biopsy, and molecular profiling provide incomplete or partially discordant information. This report illustrates how integrated interpretation of clinical context, genomic alterations, liquid biopsy findings, and tumor microenvironment features can generate molecularly informed treatment considerations for patients with difficult-to-monitor CRC. Such case-based educational discussions may help refine the use of precision oncology tools in clinical decision-making and support structured interpretation of complex molecular data in challenging clinical scenarios.
